# A set of complications during percutaneous valve-in-valve replacement: Coronary occlusion, device migration, surgical rescue, and mechanical support

**DOI:** 10.1016/j.xjse.2026.100152

**Published:** 2026-09-24

**Authors:** Catarina Relvas Novo, Mariana Campos, Belisa Gomes, António Lima, João Cardoso, Alberto Rodrigues, Paulo Neves

**Affiliations:** a Cardiothoracic Surgery Department, Unidade Local de Saúde Gaia/Espinho, Porto, Portugal; b Cardiology Department, Unidade Local de Saúde Gaia/Espinho, Porto, Portugal

**Figure:**
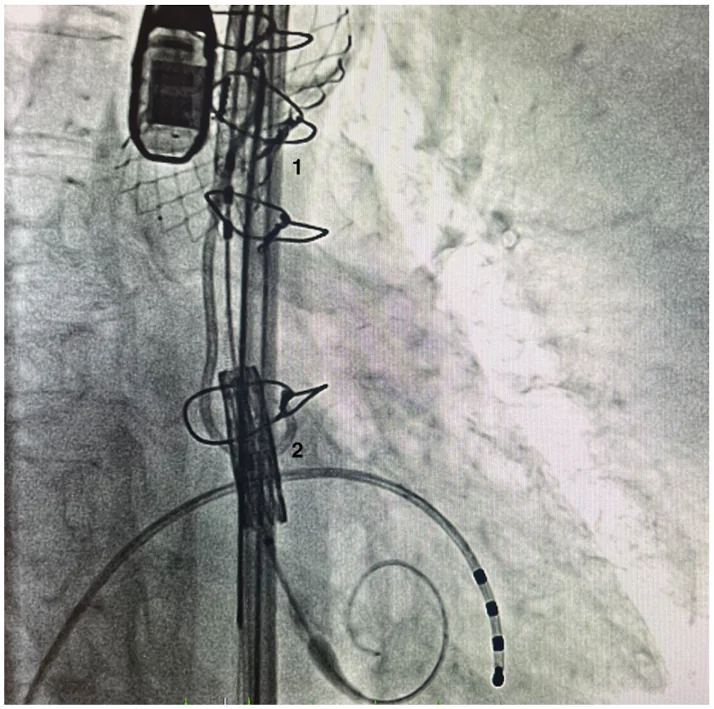
Angiography showing migrated transcatheter valve (1) and positioning of second prosthesis (2).

**Central Message**

Catastrophic valve-in-valve TAVR complications are increasing, and survival depends on rapid surgical rescue, technical versatility, and the use of advanced mechanical circulatory support.

See Discussion on page XXX.

A 75-year-old woman had undergone aortic valve replacement with a stentless bovine pericardial prosthesis 13 years before presentation. She was referred to the heart team because of severe prosthetic valve regurgitation. Her Society of Thoracic Surgeons predicted risk of morbidity and mortality was 15.6%, and her European System for Cardiac Operative Risk Evaluation II score was 8.18%. Given the extensive calcification of the aortic root and ascending aorta, with preoperative computed tomography (CT) findings suggestive of a hostile reentry, including a heavily calcified ascending aorta in close proximity to the posterior sternal table, as well as the patient's frailty and high surgical risk, after multidisciplinary heart team discussion she was selected for a percutaneous valve-in-valve procedure. Institutional review board approval was not required, because this is a single retrospective anonymized case report and does not constitute human subjects research. Written informed consent for publication was not obtained because of the retrospective nature of the report and the impracticability of obtaining consent.

Given the small surgical bioprosthesis (21 mm), a supra-annular self-expanding valve was initially selected to optimize postprocedural hemodynamics and minimize the risk of patient-prosthesis mismatch, because previous studies have demonstrated superior effective orifice areas in small valve-in-valve procedures.^1^ Furthermore, the possibility of repositioning or recapturing the device during deployment was considered an additional procedural advantage. A preprocedural CT scan showed coronary heights of 11 mm (left) and 9.5 mm (right), virtual transcatheter valve-to-coronary distance (VTC) distances of 3.6 mm (left) and 3.7 mm (right), and a valve-to-sinus-tubular junction distance (VTSTJ) distance of 2.5 mm; annular perimeter was 76.8 mm (derived diameter, 24.4 mm) and area 448.5 mm^2^ (derived diameter, 23.9 mm). Although the VTC and VTSTJ distances were borderline, the coronary heights and tubular root anatomy were judged reassuring, and BASILICA (bioprosthetic or native aortic scallop intentional laceration to prevent iatrogenic coronary artery obstruction) or other prophylactic coronary protection was not used in this case. The initial heart team assessment considered valve-in-valve transcatheter aortic valve replacement (TAVR) technically feasible, although the subsequent clinical course highlighted the limitations of current risk prediction in patients with stentless prostheses.

The implantation procedure was initially uneventful. However, immediately after deployment of the self-expanding transcatheter valve, acute left main coronary artery occlusion occurred, resulting in cardiac arrest. Cardiopulmonary resuscitation was initiated, and during attempts at coronary catheterization, the catheter became trapped within the metallic frame of the valve, causing valve migration into the ascending aorta. Spontaneous circulation was restored because valve migration relieved the coronary obstruction. After valve embolization and in the setting of ongoing torrential acute aortic regurgitation, the team considered immediate surgical conversion. However, given the patient's prohibitive surgical risk and the hemodynamic deterioration associated with severe acute aortic regurgitation, a second transcatheter valve implantation was attempted as the fastest strategy to restore valve competence.

This time, a balloon-expandable transcatheter valve was selected to avoid a similar complication; however, deployment again failed because of balloon rupture during inflation. During retrieval of the device, the valve became lodged in the right femoral artery. After a multidisciplinary heart team discussion, the patient was transferred emergently to the operating room with the percutaneous delivery system still in place.

After removal of the delivery system and the migrated valve, the right femoral artery was found to be severely damaged, requiring vascular reconstruction. Shortly after resternotomy, during cardiac mobilization and adhesiolysis, the patient experienced a second cardiac arrest and was placed on emergent central cardiopulmonary bypass with a vent through the right superior pulmonary vein. Blood cardioplegia was initially delivered retrogradely and subsequently administered directly into the coronary ostia while systemic cooling to 28 °C was achieved. A brief period of hypothermic circulatory arrest allowed safe retrieval of the migrated valve from the ascending aorta (Figure 1), with no evidence of intimal injury. The degenerated stentless prosthesis was partially explanted, with removal of the leaflets only, and a new stented aortic valve was implanted using the previous prosthetic ring as the anchoring structure for valve fixation.

**Figure 1:**
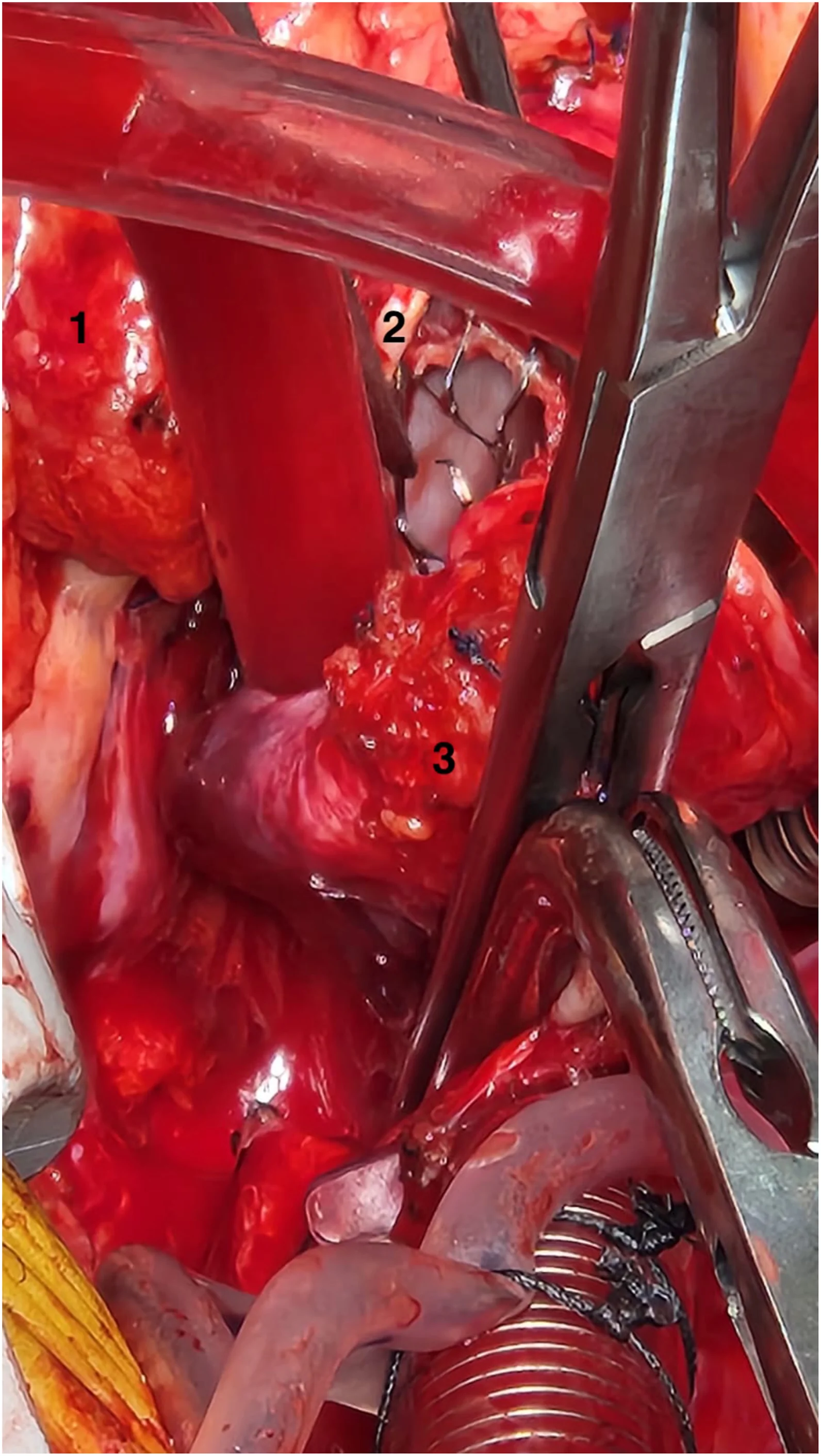
Intraoperative view of transcatheter valve retrieval from the ascending aorta under hypothermic circulatory arrest. 1, Proximal aorta. 2, Forceps removing the migrated prosthesis from ascending aorta. 3, Aortic clamp.

Weaning from cardiopulmonary bypass was difficult despite high-dose inotropic and vasopressor support, prompting the initiation of venoarterial extracorporeal membrane oxygenation (ECMO). Peripheral cannulation with surgical exploration of the femoral vessels was chosen to minimize the risk of further bleeding complications.

While the patient was receiving ECMO support, intraoperative transesophageal echocardiography revealed severe acute right ventricular dysfunction. Given the strong suspicion of right coronary artery compromise caused by the newly implanted surgical valve, and recalling the close proximity of the prosthesis to the right coronary ostium observed during implantation, the team considered coronary obstruction the most likely cause of the severe right ventricular dysfunction. Because angiography was not available in the operating room and was performed only later, confirming the diagnosis, a saphenous vein aortocoronary bypass graft was performed empirically, resulting in immediate improvement of right ventricular function.

Subsequently, progressive left ventricular dysfunction during the early postoperative period prompted implantation of an Impella CP device (J&J MedTech) via femoral access for ventricular unloading. In the catheterization laboratory, coronary angiography confirmed right coronary artery occlusion, with a patent and well-functioning saphenous vein graft supplying the affected territory, whereas the left coronary system remained unobstructed.

Over the following days, biventricular function progressively recovered. ECMO support was discontinued after 4 days, and the Impella device was removed 2 days later. The patient also required femoral artery thrombectomy and patch angioplasty reconstruction.

Postoperatively, the patient underwent intensive physical therapy and rehabilitation, demonstrating excellent recovery. She was discharged home 2 weeks after surgery and, at 1-month follow-up, remained asymptomatic with near-complete recovery of functional independence.

Freedom SOLO prostheses (Sorin Group) present a unique challenge during valve-in-valve procedures because the leaflets are mounted externally and the prosthesis is implanted in a subcoronary position, often resulting in reduced valve-to-coronary distances and an increased risk of sinus sequestration and coronary obstruction. Valve migration during valve-in-valve TAVR has been previously described and represents a particularly high-risk complication when the original aortic prosthesis is stentless.

Coronary obstruction during valve-in-valve TAVR is a rare but potentially catastrophic complication and occurs more frequently in patients with stentless prostheses or bioprostheses with externally mounted leaflets.^2^^,^^3^ No significant differences have been reported between self-expanding and balloon-expandable valves in this setting, and both types may be used when appropriate preventive strategies, such as BASILICA or chimney techniques, are used to reduce the risk of coronary obstruction.^3^ Several rescue strategies have also been reported, ranging from minimally invasive surgical approaches^4^ to implantation of a second transcatheter valve^5^ and even thoracic endovascular aortic repair rescue techniques.^E1^

Current valve-in-valve planning relies heavily on multidetector CT, which has become the cornerstone for assessment of coronary anatomy and bioprosthetic–aortic root relationships. Parameters such as coronary height, VTC distance, and VTSTJ distance are essential for predicting the risk of coronary obstruction and guiding the selective use of coronary protection strategies in high-risk anatomies.^2^^,^^E2^^,^^E3^ Importantly, this case illustrates that despite careful heart team evaluation, catastrophic coronary obstruction may still occur during valve-in-valve procedures involving stentless prostheses.

This case was particularly challenging because of the combination of multiple catastrophic complications. Not only did 2 separate valve migration events occur, but these were accompanied by coronary ostial occlusion and severe vascular injury, ultimately necessitating a highly complex redo surgical rescue procedure. To the best of our knowledge, reports describing the combination of sequential valve migration, coronary occlusion, major vascular injury, emergent redo surgery, venoarterial ECMO support, and Impella-assisted ventricular unloading are exceedingly rare.

This report highlights the challenges associated with managing catastrophic valve-in-valve TAVR complications in patients with stentless prostheses, particularly the Freedom SOLO valve, in which implantation close to the coronary ostia increases the risk of coronary obstruction.

Preprocedural CT analysis remains the cornerstone of risk assessment in valve-in-valve procedures involving stentless bioprostheses. Measurements such as coronary height, VTC, and VTSTJ should be systematically evaluated, and adjunctive techniques including BASILICA or coronary protection should be considered whenever a high risk of coronary obstruction is anticipated.

Timely recognition of procedural failure, rapid conversion to open surgery, coordinated use of advanced mechanical circulatory support, and urgent coronary revascularization were critical to achieving a favorable outcome. This case underscores the essential role of a multidisciplinary heart team and the importance of maintaining surgical readiness when performing high-risk transcatheter interventions.

### Webcast

You can watch a Webcast of this AATS meeting presentation by going to: https://www.aats.org/resources/cr3-a-perfect-storm-during-val-13338.

**Figure:**
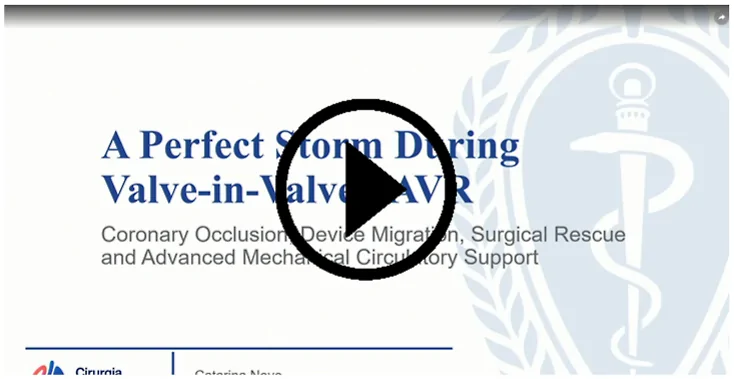
Case presentation during the 106th AATS meeting.

## Conflict of Interest Statement

The authors reported no conflicts of interest.

The *Journal* policy requires editors and reviewers to disclose conflicts of interest and to decline handling or reviewing manuscripts for which they may have a conflict of interest. The editors and reviewers of this article have no conflicts of interest.
